# Effects of acute carbohydrate ingestion on anaerobic exercise performance

**DOI:** 10.1186/s12970-016-0152-9

**Published:** 2016-11-10

**Authors:** Ben M. Krings, Jaden A. Rountree, Matthew J. McAllister, Patrick M. Cummings, Timothy J. Peterson, Brent J. Fountain, JohnEric W. Smith

**Affiliations:** 1 Department of Kinesiology, Mississippi State University, Mississippi State, MS 39762 USA; 2 Department of Food Science, Nutrition, and Health Promotion, Mississippi State University, Mississippi State, MS 39762 USA

**Keywords:** Resistance Training, Conditioning, Sprinting, Jumping

## Abstract

**Background:**

Carbohydrate (CHO) supplementation during endurance exercises has been shown to increase performance, but there is limited research with CHO supplementation during strength and conditioning exercises. Therefore, the purpose of this study was to examine the effects of various levels of CHO ingestion during acute testing sessions requiring participants to complete a strength and conditioning program designed for collegiate athletes.

**Methods:**

Participants (*n* = 7) performed a series of exercises while ingesting an amino-acid electrolyte control (CON) or CON plus varying levels of CHO. The CHO beverages delivered a 2:1 (glucose: fructose) ratio at rates of 15 g/h, 30 g/h, and 60 g/h. The exercise protocol consisted of a series of short sprints, full body resistance training exercises, jumping, and shuttle running. Performance measurements were taken for sprint times, repetitions until failure [bench press, bent over row, biceps curl, overhead triceps extension], summation of total repetitions for all repetitions until failure, repetitions in a set time for two-foot line jumps, and 137-m shuttle times.

**Results:**

A significant main effect (*p* < 0.05) was found in relation to CHO dose during the bench press final set repetitions to failure. Pairwise comparison with Bonferroni’s correction identified that there was significant difference (*p* = 0.0024) between the dosage of 15 g/h and CON during bench press. Inferential statistics identified overall RT performance with a dosage of 15 g/h compared to 60 g/h and CON was 99.2 % (very likely) and 96.7 % (very likely) to have a beneficial effect.

**Conclusions:**

The results from this study suggest acute ingestion of CHO does not result in decrements in performance and may provide a beneficial effect to strength and conditioning performance. Strength and conditioning coaches may recommend their athletes ingest CHO during training sessions in order to maximize muscular adaptations.

## Background

Ergogenic aids, including carbohydrate (CHO), are popular means of supplementation for athletes of all sports with the goal of improving performance and recovery. Strength and conditioning programs vary depending on sport, but common practices implemented in Division 1 athletics include the use of a periodization protocol, utilization of multiple sets, plyometrics training, explosive movements, and Olympic lifts [1]. While a great deal of research has investigated the role of CHO in endurance sports, CHO supplementation with resistance training (RT) has received less focus. The efficacy of CHO supplementation with a training regimen typical of full strength and conditioning sessions has received even less attention. Considering the minimal time strength and conditioning coaches are allowed to train athletes during both offseason and in-season, any enhancement of the time utilization is beneficial.

Resistance training is a large component of strength and conditioning sessions. During RT sessions requiring multiple sets with various exercises, it has been shown that muscle glycogen becomes a major fuel substrate, especially during high intensity practices. Haff et al. [2] reported a 40 % decrease in muscle glycogen stores in the vastus lateralis immediately following 39 min of lower body isokinetic and free-weight exercise. The results from this study are similar to other studies that have found significant decreases in lower body muscle glycogen stores following acute bouts of RT [3, 4]. Research examining performance with RT and CHO supplementation have shown mixed results. Haff et al. [5] reported an increase in squat performance to exhaustion during a second training sessions in the same day with CHO supplementation before, during, and after RT. Other studies have found similar increases in performance during lower body leg exercises with CHO supplemented before and during exercises [6–9]. Opposing studies have shown no increases in muscular performance following CHO supplementation before and during exercise [2, 10, 11]. These contrasting results may be related to exercise protocol, target muscle groups, and overall length of training sessions. Along with possibly increasing performance during training sessions, CHO supplementation has been shown to increase insulin levels [12, 13] and suppress protein degradation [14], increasing the anabolic environment following RT. In combination with the possibility of an acute increase in performance, CHO supplementation may provide favorable hormonal adaptations following RT.

Although RT is an important component of strength and conditioning, other facets of training such as sprints, jumps, plyometric training, and conditioning should not be overlooked. Improvements in vertical jump performance has been shown when participants are supplemented with CHO and required to repeat maximal vertical jumps [15, 16]. Contrast to the results of these published studies, results from other studies have shown CHO supplementation to have no effect on vertical jump performance [17–19]. Speed is often used as a marker for athletic success, as faster athletes tend to have greater success [20–22]. Carbohydrate supplementation and sprint performance research has shown mixed results. During soccer specific training games, a 6.4 and 6.2 % CHO solution has been shown to significantly decrease sprint time [23, 24]. When examining sprint performance during other athletic events such as simulated basketball activities, a 6 % CHO solution was shown to have no performance increases [17, 18]. With varying results on sprinting and jumping performance, more research is needed to establish the efficacy of CHO supplementation during these types of activities.

An important component of CHO supplementation is to provide optimal dosages to elicit the greatest possible ergogenic effect. The majority of CHO dose response research has been focused on endurance performance. Smith et al. [25] suggests when glucose is ingested at a rate of 15 g/h to 60 g/h during endurance exercise lasting 150 min, there is a dose-performance relationship. The optimal dosage for non-endurance activities has not been established. Studies that have shown increases in performance with CHO supplementation and RT have utilized dosages from 10 to 20 % CHO solutions [5–9] however most commerically available sports beverages are 2–8 % carbohydrate. Identifying a possible dose-performance relationship during strength and conditioning practices is an area that has received minimal research and is an important aspect in establishing the value of CHO supplementation.

Based on the current body of scientific literature, CHO supplementation with RT, jumps, and sprints have shown conflicting results. Athletes participating in multiple modes of training combined into one protocol may benefit from CHO supplementation by attenuating muscle glycogen depletion along with increasing performance. Therefore, the purpose of this investigation was to examine the effects of various levels of CHO supplementation during acute testing sessions that requires participants to complete a series of jumps, sprints, RT exercises, and shuttle runs.

## Methods

### Experimental design

The present investigation used a double-blinded, randomized, and crossover design to examine the effects of amino-acid electrolyte control (CON), in addition to CON plus varying concentrations of CHO which consisted of either 15 g/h, 30 g/h, or 60 g/h on athletic performance. The experimental period consisted of six sessions. All participants reported to the University Recreation Center following at least a 10 h fast at either 6:00, 7:30, or 9:00 AM. The first session was used to determine participants’ height (Shorrboard Measuring Device, Weigh and Measure LLC., Olney, Maryland, USA), body mass (Ironman Innerscan, Tanita, Arlington, Illinois, USA), one repetition-maximum (1-RM) of RT exercises, and familiarize participants with the protocol. The second training session served as a familiarization session with no supplementation. During testing sessions three through six, participants completed a strength and conditioning protocol while consuming either CON, CON + 15 g/h, CON + 30 g/h, and CON + 60 g/h at a 2:1 (glucose: fructose) ratio in beverage form. Participants received seven to 10 days between supplementation sessions three through six to allow for proper recovery time. During the course of the study, participants were instructed to refrain from exercising 24 h prior to sessions, but were advised to keep their normal exercise routine throughout the study and maintain normal sleep and dietary habits.

### Participants

A total of nine highly trained males volunteered to participate in the study; however, two participants withdrew during the course of the study. Therefore, data are reported for seven participants (mean ± SD; age 21.9 ± 1.6 years, body mass 91.6 ± 9.7 kg, height 181.2 ± 5.8 cm). Average daily dietary intakes during the course of data collection for participants is as follows: mean ± SD; kcal intake 2922 ± 527 kcals; CHO 46 ± 11 %; protein 30 ± 12 %; fat 26 ± 4 %. Participant inclusion criteria were as follows: (1) meet advanced level of training according to the NSCA standards [26], (2) have experience with performing hang clean and front squat exercises. In order to meet the advanced level of training according to the NSCA, participants had to have a training age of ≥ 1 year, training frequency of ≥ 3–4 days per week, high degree of training stress, and high degree of technique experience and skill [26]. All participants gave informed consent and completed a PAR-Q and general health history questionnaire in accordance with the University Institutional Review Board approval before data collection.

### Experimental procedures

#### One-repetition maximum strength testing

Maximal strength was assessed for each RT exercise by completing a predicted 1-RM test according to NSCA guidelines [26]. A total of three sets were completed for each exercise. During the first set, participants were instructed to use a weight corresponding with 50 % 1-RM for 5–10 repetitions. Following the first set and one minute of rest, participants completed a second set for 5–10 repetitions corresponding to 65–80 % 1-RM. Participants rested one minute and commenced their third and final set. During the third set, participants were instructed to perform repetitions to failure in the full exercise range of motion for 4–8 repetitions. Between each exercise participants received two minutes of rest. Load and repetitions from the final set were used to estimate 1-RM through the following formula [27]: Estimated 1-RM = Weight lifted/ (1.0278–0.278 x reps).

#### Supplementation

Participants consumed either CON, CON + 15 g/h, CON + 30 g/h, and CON + 60 g/h prior to exercise, and approximately every 15 min during exercise for a total of five ingestions. Since participants vary by the speed and time to complete jumps, sprints, and RT exercises, supplementation was given after specific exercises rather than on the exact 15 min marks. Total volume of each beverage was 118 mL and total volume consumed over the course each training session was 590 mL. Participants were only allowed to drink supplemented beverages during testing sessions. The CON treatment consisted of amino acid-electrolyte beverage mixture of 1.25 g L-Leucine, 0.625 g L-Isoleucine, 0.625 g L-Valine, 2 g L-Taurine, 1 g L-Citrulline with each serving (Dymatize Enterprises, LLC., Dallas, TX, USA). The CHO beverages consisted of CON in addition to 2:1 part (glucose:fructose) ratio consisting of delivering either 15 g/h, 30 g/h, or 60 g/h. These dosages correspond with a beverage mixture of 3, 6, and 12 % CHO. These dosages were chosen because standard sports drinks range from 6 to 8 % CHO and lower calorie produces designed for exercise occasions typically range from 2 to 3 % CHO. All treatments were manufactured by Dymatize Enterprises, LLC. The treatments were sent to a researcher who prepared beverages for the study and was excluded to all data collection and analysis. All beverages were delivered in an opaque bottle with indistinguishable flavor, taste, and color between CHO and CON treatments. Consumption of all treatments throughout the study were witnessed by the principal investigator to ensure participant compliance.

#### Testing protocol

Following a standardized dynamic warm-up, participants completed a supervised testing protocol (Table 1). The strength and conditioning protocol was designed by a Division 1 strength and conditioning coach to simulate a collegiate football training session. The overall time it took participants to complete the training protocol was 71.3 ± 2.9 min. Immediately following a dynamic warm-up, participants received the first beverage and began the protocol. During the speed work portion of the protocol, participants were instructed to jump and sprint with maximal effort. Max broad jump distance was measured and recorded in centimeters using a measuring tape (Martin Sports, Inc., Carlstedt, New Jersey, USA). All sprint exercises were electronically timed (Test Center Timing System, Brower Timing Systems, Draper, Utah, USA). Following the completion of the second 27-m sprint, the second beverage was administered and participants received two minutes of rest. Resistance training exercises were completed using the same percentage of weight based off of individual 1-RM for sessions two through six. The exercises of dumbbell (DB) bench press and barbell (BB) bent-over row were completed as a superset as was DB biceps curl and DB overhead triceps extension. During the final set of these four exercises, participants were instructed to complete as many repetitions as possible until muscular failure. The third beverage was consumed following the fifth set of front squats, the fourth was ingested following BB bent over row, and the fifth was consumed after completing DB overhead triceps extension. Following this exercise, two minutes of rest was allowed before completing the final two exercises. Performance variables used for data collection included max broad jump distance, 9-m sprint time, 18-m sprint time, 27-m sprint time, DB bench press, BB bent-over row, DB biceps curl, and DB overhead triceps extension final set repetitions, summation of total repetitions until muscular failure, repetitions in a set time for two-foot line jumps, and 137-m shuttle times. All trials were supervised by a NSCA Certified Strength and Conditioning Specialist to ensure exercises were completed with proper form and safety.

**Table 1:** Training protocol

Exercise	Intensity	Sets	Reps	Followed by seconds of rest
Speed Work				
Max Broad Jump	Max	5	1	30 s between reps
Broad Jump + 9-m Sprint	Max	4	1	45 s between reps
Overhead Medicine Ball Toss + 18-m Sprint	Max	4	1	60 s between reps
27-m Sprint	Max	2	1	60 s between reps
Resistance Training				
Hang Clean				
	50 % - Max	1	5	60 s between sets
	55 % - Max	1	4	60 s between sets
	60 % - Max	1	4	60 s between sets
	65 % - Max	1	4	60 s between sets
	70 % - Max	1	4	60 s between sets
Front Squat/Box Jump				
	45 % - Max	1	8	60 s between sets
	65 % - Max	1	5	30 s between sets
	24″ Box Jump	1	5	90 s between sets
	70 % - Max	1	5	30 s between sets
	24″ Box Jump	1	5	90 s between sets
	80 % - Max	1	3	30 s between sets
	24″ Box Jump	1	5	90 s between sets
	85 % - Max	1	3	150 s between sets
	90 % - Max	1	2	150 s between sets
	60 % - Max	1	8	180 s between sets
Dumbbell Bench Press				
	60 % - Max	1	10	60 s between sets
	65 % - Max	1	10	60 s between sets
	70 % - Max	1	10	60 s between sets
	73 % - Max	1	Failure	60 s between sets
Barbell Bent-Over Row				
	60 % - Max	1	10	60 s between sets
	65 % - Max	1	10	60 s between sets
	70 % - Max	1	10	60 s between sets
	73 % - Max	1	Failure	60 s between sets
Barbell Reverse Lunge with Front Squat Grip				
	55 % - Max	2	12	30 s between sets
	65 % - Max	2	12	30 s between sets
	70 % - Max	2	12	30 s between sets
Single Arm Shoulder Press				
	65 % - Max	2	10	30 s between sets
	70 % - Max	2	10	30 s between sets
Dumbbell Biceps Curl				
	60 % - Max	2	10	45 s between sets
	60 % - Max	1	Failure	45 s between sets
Dumbbell Overhead Triceps Extension				
	60 % - Max	2	10	45 s between sets
	60 % - Max	1	Failure	45 s between sets
Agility/Shuttle Runs				
Two Foot Line Jumps	Max	3	10 s	45 s between sets
137-m shuttle	Max	1	1	30 s between sets
137-m shuttle	Max	2	1	120 s between sets

### Statistical analysis

Data are presented as mean ± standard deviation. A significant alpha level was defined as *p* < 0.05. Data were analyzed using SAS version 9.4 (SAS Institute, Cary, NC, USA). Performance variables were analyzed using repeated measures analysis of variance (ANOVA). When significant main effects were found, a Tukey Post-Hoc was performed with a Bonferroni correction to further investigate differences and to compare means between doses.

Prior to beginning data collection, the decision was made to also analyze the data using inferential statistics. To allow for the use of inferential statistics, meaningful effect based inferences as described by Hopkins et al. [28], pairwise t-tests were performed comparing individual dosages. A probabilistic magnitude-based inferential analysis was conducted with each comparison to determine the likelihood of a performance enhancement between CHO ingestion rates. Based on data reported by Hopkins et al. [29] that the smallest meaningful effects in athletes is ~0.3–0.7 of the coefficient of variation, this study used 0.5 of the coefficient of variation as the smallest meaningful improvement. Confidence intervals of 90 % defined the uncertainty of the measure. The qualitative descriptions describing the chances the findings are larger than the smallest meaningful effect (0.5 X coefficient of variation) are: < 0.5 %, most unlikely or almost certainly not; 0.5–5 %, very unlikely; 5–25 %, unlikely or probably not; 25–75 %, possibly; 75–95 %, likely or probably; 95–99.5 %, very likely; > 99.5 %, most likely or almost certainly [30].

Intraclass correlation coefficients (ICC) were calculated to predict reliability for all dependent variables. McGraw and Wong [31] model (1, k) was used to calculate ICC. Cohen’s effect size and sample size needed for a magnitude-based inference about the practical significance of the observed changes in performance for a power of 80 % were also calculated.

## Results

A significant main effect was found in relation to CHO dose during the DB bench press final set to failure. All carbohydrate treatments improved performance in comparison to the non-carbohydrate CON during DB bench press. However, after a Bonferroni correction the comparison of CON to 15 g/h was the only comparison that was significantly different (*p =* 0.0024). The pairwise t-tests (conducted for between treatments for meaningful effect inferences) demonstrated a significant improvement in performance when 60 g/h was consumed during the 27-m sprint as compared to CON. Additionally, these pairwise t-tests demonstrated 15 g/h resulted in significant improvements in performance in summation of total repetitions performed as compared to 60 g/h.

Table 2 presents results from DB bench press, BB bent-over row, biceps curl, and overhead triceps extension. When compared to the CON, all three CHO dosages significantly improved performance (Table 2) during DB bench press. However, a 95 % likelihood for performance improvement was seen with 15 g/h compared to 60 g/h. During BB bent-over row, 15 g/h and 30 g/h demonstrated a likely improvement in performance compared to both CON and 60 g/h. The dosage of 30 g/h yielded the greatest performance during biceps curl. Chances of an increase in performance were very likely (95.4 %). Performance was shown to likely decrease when comparing 60 g/h to 30 g/h (-94.7 %). Inferential statistics demonstrate minimal increases in performance with all three dosages during overhead triceps extension.

**Table 2:** Performance during resistance training exercises

		Total reps	15 g/h	30 g/h	60 g/h
Bench Press	CON	9.7 ± 2.9	27.9 %; 1.3 to 4.1 (0.83) 99.4 %, very likely (4) *p* = 0.01	20.6 %; 0.7 to 3.3 (0.72) 98.2 %, very likely (6) *p* = 0.03	20.6 %; 0.5 to 3.5 (0.66) 97.5 %, very likely (7) *p* = 0.04
	15 g/h	12.4 ± 3.6		-5.8 %; -2.0 to 0.6 (0.22) 78.9 %, likely (63) *p* = 0.33	-5.8 %; -1.3 to -0.2 (0.21) -95.5 %, very likely (7) *p =* 0.05
	30 g/h	11.7 ± 2.7			No differences n/a *p* = 1.00
	60 g/h	11.7 ± 3.2			
Bent-Over Row	CON	16.6 ± 5.3	6.9 %; -2.6 to 4.9 (0.18) 68.4 %, possibly (157) *p* = 0.57	6.0 %; -1.28 to 3.8 (0.20) 71.5 %, possibly (108) *p* = 0.51	-7.7 %; -3.3 to 0.7 (0.28) -84.9 %, likely (35) *p* = 0.25
	15 g/h	17.7 ± 7.2		-0.8 %; -3.7 to 3.4 (0.02) 43.5 %, possibly (30886) *p* = 0.94	-13.7 %; -6.3 to 1.4 (0.42) -85.0 %, likely (24) *p =* 0.27
	30 g/h	17.6 ± 4.5			-13.0 %; -4.2 to -0.31 (0.56) -96.1 %, very likely (9) *p* = 0.07
	60 g/h	15.3 ± 3.7			
Biceps Curl	CON	12.3 ± 4.9	3.5 %; -0.8 to 1.7 (0.09) 64.0 %, possibly (528) *p* = 0.53	16.3 %; 0.3 to 3.8 (0.37) 95.4 %, very likely (10) *p* = 0.07	-5.8 %; -2.3 to 0.9 (0.15) 16.0 %, unlikely (157) *p* = 0.43
	15 g/h	12.7 ± 4.8		-12.4 %; -1.1 to 4.3﻿﻿ (0.29)﻿ ﻿ 82.0 %, likely (39) *p* = 0.30	-9.0 %; -2.9 to 0.6 (0.24) -84.0 %, likely (41) *p =* 0.24
	30 g/h	14.3 ± 5.9			-19.0 %; -5.8 to -0.14 (0.52) -94.7 %, likely (11) *p =* 0.09
	60 g/h	11.6 ± 4.6			
Overhead Triceps Extension	CON	13.7 ± 4.5	7.3 %; -1.1 to 3.1 (0.21) 76.3 %, likely (80) *p* = 0.39	2.1 %; -2.0 to 2.5 (0.07) 54.5 %, possibly (1659) *p* = 0.81	-1.0 %; -2.0 to 1.7 (0.03) 37.0 %, possibly (10992) *p* = 0.88
	15 g/h	14.7 ± 5.2		-4.9 %; -3.6 to 2.2 (0.15) 28.8 %, possibly (261) *p* = 0.65	-4.9 %; -3.6 to 2.2 (0.15) 28.8 %, possibly (261) *p* = 0.65
	30 g/h	14.0 ± 4.2			-3.1 %; -3.8 to 3.0 (0.08) 37.13 %, possibly (1308) *p =* 0.82
	60 g/h	13.6 ± 5.8			

Data indicate total repetitions for all RT exercises with ingestion of 15, 30, and 60 g/h of 2:1 glucose: fructose (mean total repetitions ± SD) and %improvement in total repetitions [1st line: %improvement, 90 % confidence interval limits, and Cohen’s effect size (ES; in parentheses); 2nd line: chances (% and qualitative) of meaningful improvement (>0.5 % CV) and sample size needed for a magnitude-based inference about the practical significance of the observed changes in performance for a power of 80 % (in parentheses); 3rd line: exact *P* value from pairwise t-tests]

A summation of total repetitions from all RT exercises is presented in Table 3. The dosage of 15 g/h had the greatest performance when compared to CON (96.7 %, very likely), and approached significance (*p =* 0.06) with the pairwise t-tests used during inferential analysis. Performance with 60 g/h was significantly lower (*p* = 0.01) than 15 g/h with the same t-tests. Inferences suggest that 15 g/h is 99.2 % (very likely) to improve performance compared to 60 g/h. CHO supplementation of 30 g/h had the likelihood to improve performance when compared to CON, and 60 g/h was likely to negatively impact performance when compared to 30 g/h. Dosage rates of 15 g/h and 30 g/h had the greatest likelihood to improve performance when repetitions from DB bench press, BB bent-over row, biceps curl, and overhead triceps extension were summated.

**Table 3:** Total repetitions performance

		Total reps	15 g/h	30 g/h	60 g/h
Total Repetitions	CON	52.3 ± 14.2	10.1 %; 0.8 to 9.8 (0.42) 96.7 %, very likely (9) *p* = 0.06	10.1 %; -1.2 to 11.8 (0.39) 91.2 %, likely (19) *p* = 0.17	-0.3 %; -3.5 to 3.2 (0.01) 44.1 %, possibly (123526) *p* = 0.94
	15 g/h	57.6 ± 10.5		No differences n/a *p* = 1.00	-9.4 %; -8.5 to -2.3 (0.47) -99.2 %, very likely (2) *p =* 0.01
	30 g/h	57.6 ± 13.2			-9.4 %; -13.3 to 2.41 (0.42) -88.2 %, likely (21) *p =* 0.23
	60 g/h	52.1 ± 12.6			

Data indicate summation of total repetitions for all RT exercises with ingestion of 15, 30, and 60 g/h of 2:1 glucose: fructose (mean total repetitions ± SD) and %improvement in total repetitions [1st line: %improvement, 90 % confidence interval limits, and Cohen’s effect size (ES; in parentheses); 2nd line: chances (% and qualitative) of meaningful improvement (>0.5 % CV) and sample size needed for a magnitude-based inference about the practical significance of the observed changes in performance for a power of 80 % (in parentheses); 3^rd^ line: exact *P* value from pairwise t-tests]

Performance during sprints and shuttle runs is represented in Table 4. Using the pairwise t-tests for inferential statistics, there was significant reduction in sprint time (*p* = 0.04), with 60 g/h compared to CON during 27-m sprint time. The significant reduction in sprint time at 60 g/h corresponds with a very likely (96 %) increase in performance. At a dosage of 30 g/h, performance was likely to improve, compared to CON. During 18-m sprints, 60 g/h was the only dosage to have a likelihood to improve performance when compared to CON. Performance did not improve at all three dosages when compared to CON during 9-m sprints. A dosage of 30 g/h was likely (90.1 %) to improve 127-m shuttle performance when compared to 15 g/h. Inferences also suggest 30 g/h has a likely (76.4 %) chance to improve performance compared to CON. Carbohydrate supplementation of 60 g/h had the greatest performance during 27-m and 18-m sprints compared to 15 g/h, 30 g/h, and CON.

**Table 4:** Performance during sprinting and shuttle runs

		Time (sec)	15 g/h	30 g/h	60 g/h
27-m Sprints	CON	4.26 ± 0.15	-0.3 %; -0.12 to 0.09 (0.06) 42.9 %, possibly (2374) *p* = 0.83	-1.3 %; -0.14 to 0.03 (0.42) 80.1 %, likely (23) *p* = 0.25	-1.9 %; -0.14 to -0.02 (0.56) 96.0 %, very likely (7) *p* = 0.04
	15 g/h	4.25 ± 0.24		-1.0 %; -0.20 to 0.12 (0.23) 60.2 %, possibly (93) *p* = 0.62	-1.7 %; -0.16 to 0.01 (0.36) 84.5 %, likely (19) *p =* 0.16
	30 g/h	4.21 ± 0.11			-0.7; -0.10 to 0.05 (0.22) 62.6 %, likely (98) *p =* 0.51
	60 g/h	4.18 ± 0.14			
18-m Sprints	CON	2.95 ± 0.09	0.2 %; -0.06 to 0.07 (0.05) 23.4 %, unlikely (1980) *p* = 0.86	0.5 %; -0.09 to 0.12 (0.12) 27.6 %, possibly (662) *p* = 0.79	-1.4 %; -0.10 to 0.02 (0.48) 78.77 %, likely (113) *p* = 0.23
	15 g/h	2.96 ± 0.15		0.3 %; -0.12 to 0.14 (0.06) 31.7 %, possibly (3099) *p* = 0.90	0.3 %; -0.11 to 0.13 (0.06) 31.3 %, possibly (3099) *p =* 0.90
	30 g/h	2.97 ± 0.14			-1.9 %; -0.14 to 0.03 (0.49) 82.6 %, likely (22) *p =* 0.23
	60 g/h	2.91 ± 0.08			
9-m Sprints	CON	1.43 ± 0.09	0.8 %; -0.03 to 0.05 (0.14) 5.1 %, unlikely (255) *p* = 0.58	0.6 %; -0.02 to 0.03 (0.11) 2.0 %, very unlikely (358) *p* = 0.53	0.2 %; -0.04 to 0.1 (0.04) 16.8 %, unlikely (6570) *p* = 0.90
	15 g/h	1.44 ± 0.08		-0.2 %; -0.04 to 0.04 (0.04) 11.3 %, unlikely (6349) *p* = 0.89	-0.6 %; -0.05 to 0.04 (0.12) 26.9 %, possibly (465) *p =* 0.72
	30 g/h	1.44 ± 0.07			-0.4 %; -0.03 to 0.02 (.09) 12.0 %, unlikely (696) *p =* 0.65
	60 g/h	1.43 ± 0.07			
137-m Shuttle Runs	CON	39.8 ± 4.01	-0.2 %; -2.0 to 1.8 (0.03) 44.7 %, possibly (13118) *p* = 0.93	-2.3 %; -3.1to 1.3 (0.24) 76.4 %, likely (74) *p* = 0.45	-1.7 %; -3.0 to 2.4 (0.19) 68.5 %, possibly (155) *p* = 0.61
	15 g/h	39.7 ± 3.12		-2.1 %; -1.9 to 0.2 (0.24) 90.1 %, likely (31) *p* = 0.18	-1.4 %; -1.7 to 0.6 (0.20) 80.1 %%, likely (82) *p =* 0.37
	30 g/h	38.9 ± 3.66			0.7 %; -0.9 to 1.4 (0.08) 31.8 %, possibly (936) *p =* 0.68
	60 g/h	39.2 ± 2.65			

Data indicate sprinting times with ingestion of 15, 30, and 60 g/h of 2:1 glucose: fructose (mean total repetitions ± SD) and %improvement in sprinting time [1st line: %improvement, 90 % confidence interval limits, and Cohen’s effect size (ES; in parentheses); 2nd line: chances (% and qualitative) of meaningful improvement (>0.5 % CV) and sample size needed for a magnitude-based inference about the practical significance of the observed changes in performance for a power of 80 % (in parentheses); 3^rd^ line: exact *P* value from pairwise t-tests]

Table 5 represents performance during max broad jumps. The greatest likelihood to improve performance was seen when 60 g/h (81.7 %) was compared to 30 g/h. Performance was likely negatively impacted (-79.9 %) when comparing 30 g/h to 15 g/h. Table 6 represents total touches during two-foot line jumps. All three CHO dosages were likely to improve performance compared to CON.

**Table 5:** Performance during max broad jumps

		Average distance (cm)	15 g/h	30 g/h	60 g/h
Max Broad Jumps	CON	246.2 ± 29.7	1.0 %; -5.0 to 10.0 (0.09) 72.3 %, possibly (553) *p* = 0.55	-1.2 %; -13.3 to 7.2 (0.12) 28.8 %, possibly (341) *p* = 0.58	0.7 %; -8.7 to 12.1 (0.06) 61.7 %, possibly (1987) *p* = 0.76
	15 g/h	248.7 ± 26.6		2.2 %; -17.4 to 6.3 (0.22) -79.9 %, (likely) (76) *p* = 0.40	-0.3 %; -9.8 to 8.3 (0.03) 43.3 %, possibly (10586) *p =* 0.87
	30 g/h	243.2 ± 22.6			2.0 %; -4.6 to 14.2 (0.21) 81.7 %, likely (74) *p =* 0.68
	60 g/h	247.2 ± 29.7			

Data indicate max broad jump distance with ingestion of 15, 30, and 60 g/h 2:1 glucose: fructose (mean total repetitions ± SD) and %improvement in jump distance [1st line: %improvement, 90 % confidence interval limits, and Cohen’s effect size (ES; in parentheses); 2nd line: chances (% and qualitative) of meaningful improvement (>0.5 % CV) and sample size needed for a magnitude-based inference about the practical significance of the observed changes in performance for a power of 80 % (in parentheses); 3^rd^ line: exact *P* value from pairwise *t*-test]

**Table 6:** Performance during two-foot line jumps

		Total touches	15 g/h	30 g/h	60 g/h
2 ft Line Jumps	CON	27.9 ± 2.1	3.4 %; -0.1 to 2.0 (0.39) 92.4 %, likely (16) *p* = 0.14	3.4 %; -0.1 to 2.0 (0.39) 92.4 %, likely (16) *p* = 0.14	2.7 %; -1.0 to 2.5 (0.25) 77.4 %, likely (65) *p* = 0.42
	15 g/h	28.8 ± 2.7		-1.3 %; -1.3 to 0.6 (0.14) 21.1 %, unlikely (197) *p* = 0.47	-0.7 %; -2.1 to 1.7 (0.06) 40.4 %, possibly (2511) *p =* 0.85
	30 g/h	28.4 ± 2.8			0.7 %; -1.2 to 1.6 (0.06) 57.0 %, possibly (2194) *p =* 0.80
	60 g/h	28.6 ± 3.7			

Data indicate total touches with ingestion of 15, 30, and 60 g/h of 2:1 glucose: fructose (mean total repetitions ± SD) and %improvement in total touches [1st line: %improvement, 90 % confidence interval limits, and Cohen’s effect size (ES; in parentheses); 2nd line: chances (% and qualitative) of meaningful improvement (>0.5 % CV) and sample size needed for a magnitude-based inference about the practical significance of the observed changes in performance for a power of 80 % (in parentheses); 3^rd^ line: exact *P* value from pairwise t-tests]

Calculated ICC values for all dependent variable are as follows: DB bench press (0.72), BB bent-over row (0.89), overhead triceps extension (0.97), biceps curls (0.89), total repetitions (0.88), max broad jump (0.98), 9-m sprint (0.98), 18-m sprint (0.87), 27-m sprint (0.87), two-foot line jumps (0.95), and 137-m shuttle (0.96).

## Discussion

Based on the combined findings of this study across strength and conditioning exercises, ingestion of CHO has a likelihood to improve performance compared to an amino-acid electrolyte beverage that did not contain CHO. The likelihood of an amino-acid electrolyte beverage to lead to an acute improvement in performance as compared to a CHO containing beverage is small. Previous research has demonstrated no ergogenic effect with an amino-acid beverage compared to a placebo when completing a series of sprints, jumps, and resistance training exercises [32]. Findings from the present investigation show similar results compared to previous studies reporting improvements in RT performance with CHO supplementation [5–9]. Along with increases in bench press performance, magnitude based inferential statistical analysis found that CHO significantly increased 27-m sprint time and overall RT performance, measured by summation of total repetitions over the last set of each RT exercise. Based off of the results from both traditional statistics and magnitude based inferences, CHO supplementation demonstrated performance improvement during multiple aspects of a traditional collegiate strength and conditioning protocol.

Investigations reporting no increases in RT performance with CHO supplementation may be related to the inability to sensitively measure changes based off of repetition count, overall exercise protocol, CHO dosages, and exercise intensity. Kulik et al. [11] reported no increases with CHO during 5 sets of back squat at 85 % 1-RM until exhaustion. The overall protocol lasted approximately 29 min compared to the present investigation length of a 71 min. Other studies reporting similar findings, also employed shorter duration exercise protocols [2, 10]. The longer duration of the present investigation may partially explain ergogenic effects of CHO. It is well known that muscle glycogen depletion increases with prolonged and high intensity RT [3, 33–35]. As muscle glycogen depletes during a protocol that requires participants to complete RT exercise, maximal sprints, repeated maximal jumps, and shuttle runs, exogenous glucose may spare glycogenolysis and become a preferred fuel. A unique aspect of the RT protocol of the present investigation is the use of upper body exercise completed until muscular failure. A majority of previous research has been focused primarily on CHO supplementation to lower body exercises [2, 5–9, 11]. DB bench press was the only dependent measure that elicited a significant main effect and after a Bonferroni correction was applied, performance was significantly increased when 15 g/h of CHO was administered compared to CON. Therefore, caution is warranted when making strong interpretations from these data. Although traditional statistics are common place in strength and conditioning research, the decision was made a priori to also analyze results using magnitude based inferential statistics. Due to the large percentage changes necessary to assess changes in performance through repetition count based off of traditional statistical analyses alone, inferential statistics provide a meaningful interpretation to strength and conditioning coaches. Based off our results, we were able to determine that CHO ingestion overall appears to be more beneficial from an ergogenic standpoint. This is apparent especially since a small ergogenic effect may be smaller than what can be determined by traditional statistical analysis. Summation of total repetitions until failure with 15 g/h of CHO compared to 60 g/h and CON was 99.2 % (very likely) and 96.7 % (very likely) to have a beneficial effect, respectively. The greater physiological demands from the high intensity/volume full body exercise protocol may possibly explain the increase in performance during a final set until failure during DB bench press and overall summation of repetitions with 15 g/h of CHO. In addition, due to the overall protocol length, the ergogenic effect elicited to RT performance with exogenous CHO supplementation may be a product of volume completed within a training session.

Literature indicating decreases in sprint time during soccer specific drills [23, 24] and increases in jump height [15, 16] have employed varying exercise protocols, making it is difficult to directly compare results of the present investigation to previous studies. In the current investigation, participants completed maximal jumps and short sprints within the first 15 min of the exercise protocol in which they only received one beverage. Inferential statistics suggested that a dosage of 60 g/h significantly improved performance compared to CON during the 27-m sprints. A possible method for a significant performance benefit from only supplementation of 60 g/h may possibly be explained through the benefits of CHO mouth rinse. In a recent review, Jeukendrup and Chambers [36] indicate that CHO may improve performance through non metabolic means. The mechanisms include stimulation of the positive afferent signals, modifying motor output and increasing performance. The mouth rinse technique has been shown to increase performance during endurance activities [37–40], but to have no significant changes in performance when completing repeated sprints [41], muscular strength and muscular endurance [42, 43]. However, additional research with CHO mouth rinse during short duration maximal sprints, and jumps is needed to examine the possible ergogenic effects of CHO using this mechanism.

When comparing doses of CHO, the separation on performance enhancement during a strength and conditioning protocol becomes difficult to discern. Performance was found to improve significantly for one exercise at a dose that it did not improve for another. A dose-performance relationship may exist during endurance exercise [25, 44], but results from the current investigation make it difficult to discern between dosages for strength and conditioning exercises. While not measured, this could possibly be explained due to the differing physical demands between exercises and potentially due to the ability of the gut to empty its contents versus increased beverage volumes remaining in the stomach or intestine.

There are several limitations to our study. Although average dietary intakes from the participants was measured throughout the duration of the present investigation, dietary information 24 h prior to experimental testing sessions was not monitored. Although participants were instructed to maintain similar dietary intakes for the day leading up to each acute training session, muscle glycogen stores leading into training days could be affected by diet. Second, there was no true placebo administered. With no true placebo, comparisons cannot be made between the effects of CHO and CON supplementation to no supplement. Another limitation is the lack of mechanistic data collection such as blood markers. Although we lacked mechanistic data, results from this study indicate the possibility of performance increments with CHO supplementation during strength and conditioning training. Future studies in this area should focus on mechanistic data collection to analyze muscle metabolism and markers of muscle damage, to possibly explain the results of this current study.

## Conclusions

The data from this investigation indicate that CHO supplementation has an increased likelihood to improve performance compared to non-CHO when completing an acute strength and conditioning protocol. Although there were significant main effects found in relation to DB bench press performance and performance was very likely to increase with 15 g/h in overall RT performance and 60 g/h in 27-m sprint performance, there were also RT, jumping, and sprinting performance variables that suggested slight beneficial effects. Without measurement of mechanistic data as previously explained, it is difficult to explain the possible mechanisms why one exercise was significantly improved and others were not. Future studies are needed to further investigate the efficacy of CHO ingestion during strength and conditioning training along with focusing on the effects of CHO supplementation compared to a true placebo. Overall, the combined results of this study would suggest that CHO ingestion rates of 15–30 g/h with ~500 mL of fluid will likely lead to the greatest overall performance compared to supplementing only amino acids during acute strength and conditioning training sessions.

## Abbreviations

1-RM: One- repetition maximum
ANOVA: Analysis of variance
BB: Barbell
CHO: Carbohydrate
CON: Amino-acid electrolyte control
DB: Dumbbell
RT: Resistance training
